# Rapid Implementation of a Community–Academic Partnership Model to Promote COVID-19 Vaccine Equity within Racially and Ethnically Minoritized Communities

**DOI:** 10.3390/vaccines10081364

**Published:** 2022-08-20

**Authors:** Jacinda C. Abdul-Mutakabbir, Cristie Granillo, Bridgette Peteet, Alex Dubov, Susanne B. Montgomery, Jasmine Hutchinson, Samuel Casey, Kelvin Simmons, Alex Fajardo, Juan Carlos Belliard

**Affiliations:** 1 Department of Pharmacy Practice, School of Pharmacy, Loma Linda University, Loma Linda, CA 92350, USA; 2 Department of Basic Sciences, School of Medicine, Loma Linda University, Loma Linda, CA 92350, USA; 3 Institute for Community Partnerships, Loma Linda University Health, Loma Linda, CA 92350, USA; 4 Department of Psychology, School of Behavioral Health, Loma Linda University, Loma Linda, CA 92350, USA; 5 Inland Empire Concerned African American Churches, San Bernardino, CA 92411, USA; 6 Congregations Organized for Prophetic Engagement, San Bernardino, CA 92411, USA; 7 El Sol Neighborhood Educational Center, San Bernardino, CA 92410, USA; 8 Department of Global Health, School of Public Health, Loma Linda University, Loma Linda, CA 92350, USA

**Keywords:** racially and ethnically minoritized groups, COVID-19 vaccinations, health equity, vaccine equity

## Abstract

Coronavirus disease 2019 (COVID-19) has highlighted inequities in mortalities and associated illnesses among non-Hispanic Black and Hispanic/Latino individuals. Immunization against COVID-19 is critical to ending the pandemic, especially within racial and ethnically minoritized communities. However, vaccine hesitancy and institutional mistrust in these communities, resulting from decades of mistreatment, structural racism, and barriers to vaccination access, have translated into low vaccination uptake. Trustworthy relationships with healthcare professionals and partnerships with faith and community leaders are critical to increasing vaccination rates within these minoritized communities. Loma Linda University researchers collaborated with local faith and community organizations in San Bernardino County, CA, to rapidly implement a three-tiered approach to increase the vaccination rates within non-Hispanic Black and Hispanic/Latino communities. This community–academic partnership model provided over 1700 doses of the COVID-19 vaccine within these vaccine-hesitant, targeted minoritized communities. As over 100,000 individuals are diagnosed with COVID-19 daily and updated vaccines targeting variants of the Omicron strain are expected to rollout in the coming months, the development of sustainable programs aimed at increasing vaccine uptake within vulnerable communities are of the utmost importance.

## 1. Introduction

In March 2020, coronavirus disease 2019 (COVID-19) was declared a global pandemic [1,2]. By July 2022, over one million deaths were recorded in the Unites States [3,4]. Moreover, the COVID-19 pandemic has caused disproportionate harm to racially and ethnically minoritized groups, including non-Hispanic Black and Latino/Hispanic communities [5,6,7]. Individuals from these communities are 1.6 times more likely to be infected, 2.5 times more likely to be hospitalized, and 2.1 times more likely to die of COVID-19 than White Americans [8]. The overrepresentation of minoritized communities in COVID-19 deaths and hospitalization rates can be attributed to existing structural and social determinants of health inequities among these groups [6,9]. These inequities include their increased likelihood of occupying essential worker roles (“blue-collar” positions) [6,10], that consistently place them at risk of severe acute respiratory syndrome coronavirus virus 2 (SARS-CoV-2) exposure and/or transmission. In addition, unjust housing policies have resulted in higher density living situations for minoritized communities with fewer opportunities to isolate and quarantine [6,10].

The US is well-positioned to overcome the pandemic with the US Food and Drug Administration (FDA) approval of three vaccines against COVID-19, including the two-dose mRNA vaccines (developed by Pfizer^®^ (New York, NY, USA) and Moderna^®^ (Cambridge, MA, USA)) and the single-dose, viral-vectored vaccine (developed by Johnson and Johnson^®^ (New Brunswick, NJ, USA); Janssen^®^ (Beerse, Belgium)) [11,12,13]. Despite being more affected, non-Hispanic Black and Hispanic/Latino individuals are less likely to be vaccinated against COVID-19 [14]. Two years into the pandemic and one year into the availability of vaccines, vaccine hesitancy and unequal access are the two factors used to explain the lower vaccination rates among minoritized communities [15,16]. Vaccine hesitancy, defined as “delay in acceptance or refusal of vaccines despite availability,” results from decades of medical mistreatment of minoritized groups by the US healthcare system [6,16,17,18]. Multiple structural barriers hinder the ability of individuals from minoritized communities to access vaccination [6,19,20,21]. For instance, there is a lack of racially concordant, trustworthy messengers dedicated to dispelling misinformation propagated across racially and ethnically minoritized groups [16]. Additionally, traditional clinic schedules in primary care complicate the ability of minoritized and “blue-collar” individuals to receive vaccination within the typical time frames in which they are provided (9 a.m.–5 p.m. hours) [9,10]. Finally, digital and transportation inequities, experienced more by minoritized groups, have also influenced access to vaccination information and services [22].

Religious beliefs may serve as an additional barrier to immunization among minoritized groups [23,24]. Several studies suggest that some elements of vaccine hesitancy among minoritized communities might be rooted in faith; thus, faith-based approaches are important to help address such concerns and improve vaccine confidence and acceptance [24]. With this, the trusting infrastructure developed by religious leaders may be pivotal in promoting immunizations in minoritized communities [23,24]. As such, community–academic partnerships that include faith leaders—as well as other trusted messengers identified by them—are critical to increasing vaccination uptake [17,24].

## 2. Implementation Methods and Design

In San Bernardino County, CA, the largest county in the US and one of the nation’s most populous, COVID-19 case rates have outpaced state and national rates [4,25]. Moreover, non-Hispanic Black and Hispanic/Latino persons have been more likely to die of COVID-19 than most other racial and ethnic groups [3,4,26]. In early 2021, at the height of the COVID-19 pandemic, Loma Linda University (LLU) facilitated an extensive mass vaccination clinic in San Bernardino County [17]. Although an important effort that reached large numbers of residents, this mass vaccination clinic was located in a suburban setting with minimal access to public transit. It also relied on patient registration using a web-based application [17]. In February 2021, one month following the rollout of this vaccination clinic, non-Hispanic Black and Hispanic/Latino individuals accounted for only 20% of the total vaccines given, severely disproportionate to the burden of the disease among these groups and regional demographics [17]. Realizing this gap, we rapidly implemented a community–academic, three-tiered approach to increase vaccination rates within the non-Hispanic Black and Hispanic/Latino communities of San Bernardino County, CA [17].

### 2.1. Application of Three-Tiered Approach

This equity-focused approach to increase vaccine uptake was initially developed to reach our non-Hispanic Black communities [17]. We then adapted and expanded it to engage Hispanic/Latino communities [27].

*Tier 1: The Engagement of non-Hispanic Black and Hispanic/Latino Faith and Community Leaders*

The first tier included leveraging the university’s long-standing relationships to develop and execute COVID-19 community vaccination clinics [17]. LLU has had a long-standing relationship with two faith- based organizations: Congregations Organized for Prophetic Engagement (COPE) and the Inland Empire Concerned African American Churches (IECAAC) [17]. We also partnered with a Hispanic/Latino organization led by community health workers, El Sol Neighborhood Educational Center (El Sol), to engage Hispanic/Latino faith and community leaders. The first step in building the partnerships with the leaders of the organizations included identifying key faculty members to be involved in the partnership and scheduling weekly meetings to collaborate with the religious and community leaders. It is important to note that the interdisciplinary university faculty (pharmacy, public health, and behavioral health) were racially and ethnically concordant, and most had previously worked in partnership with the communities.

Community partners COPE, IECAAC, and El Sol identified major barriers to vaccination within their respective communities. The barriers included fear and mistrust regarding the vaccine components and development timeline, misinformation leading to a disbelief in the COVID-19 pandemic severity and its impact on the health of minoritized individuals, as well as the overall inaccessibility of COVID-19 vaccinations. To overcome barriers related to misinformation, a non-Hispanic Black pharmacist trained in infectious diseases and public health, presented clinical trial data for each of the FDA-approved COVID-19 vaccines to community members. The pharmacist’s presentation included a special focus on the inclusion of racially and ethnically minoritized groups and elderly individuals in the clinical trials to emphasize the vested interest in evaluating those individuals represented disproportionately amongst COVID-19 mortality rates. The community partners then scheduled a series of educational webinars to be offered before each vaccination clinic to build community trust and confidence in the vaccine. The presentations were translated and made available in Spanish given the high proportion of Spanish speaking residents.

*Tier 2: COVID-19 Educational Webinars hosted by Faith Organizations and Racially Concordant Faculty*

The second tier was the completion of educational webinars (each one hour in length), utilizing the faith-based organizations’ web-based infrastructure [28]. Faith and/or community leaders facilitated each webinar. To represent the targeted racial groups, the webinars were led by non-Hispanic Black and Hispanic/Latino LLU faculty members, with relevant professional expertise. The webinars focused on the impact of COVID-19 on minoritized groups, described the psychology related to vaccine hesitancy and fear, and translated the clinical studies to explicitly describe the vaccines’ safety and efficacy to confront the aforementioned vaccination barriers. Peteet et al. has previously described the webinar aspect of the community–academic partnership in extensive detail [28,29].

*Tier 3: Low-barrier Community Vaccination Clinics Located at the Churches of the Participating Faith Leaders*

The third tier included the implementation of low-barrier community vaccination clinics [17]. The clinics sought to address the barriers to COVID-19 vaccinations that had been identified by our community partners within their respective minoritized communities: increasing COVID-19 vaccine allocation and providing easier access within communities heavily populated by minoritized groups; providing transportation to the vaccination clinics organized by each congregations (walk-in, non-digitized registration); and participation by racially concordant clinicians who took time to further engage and discuss participants’ questions.

### 2.2. Selection of Community Vaccination Clinic Locations

Faith and community leaders selected centrally located sites that would serve as the vaccination locations based on the needs of their communities. The clinics were conducted weekly and alternated between majority non-Hispanic Black and Hispanic/Latino communities. The community vaccination clinics were held in urban and rural locations, easily accessible to the targeted vaccinees and churches, which encouraged members to provide transport.

A member of either the COPE or El Sol (depending on targeted group, non-Hispanic Black or Hispanic/Latino) leadership teams worked collaboratively with a selected team member from the LLU Institute for Community Partnerships (ICP) to develop promotional material and identify the logistical needs for implementing each vaccination clinic. The church sites allocated an area on their campus to serve as the vaccinating stations and provided chairs and tables needed for the vaccination activities. The clinics were held on either a Friday or Saturday to allow the participants’ ease in attending, such as avoiding work conflicts. For the clinics held on the weekday, the concluding times were set after working hours (6 pm and later) to allow individuals with stringent employment obligations to attend without missing work.

### 2.3. Community Vaccination Clinic Registration

During the February and March 2021 vaccine clinics—when vaccine demand exceeded supply—the COPE and El Sol team leaders distributed pre-numbered, paper-based, vaccine consent forms to local community members via email. This approach ensured that vaccines were only accessible to the intended minoritized communities. The faith and community leaders also played an important role in identifying higher-risk seniors and helping to organize transportation for them. When vaccine supply matched the demand, COPE and El Sol team leaders created pre-registration sign-up links and disseminated them amongst their communities and webinar participants, allowing for preliminary clinic turnout estimates. We also provided the paper-based registration forms to use for on-site registration, for those patients without the ability to pre-register. The registration forms included the necessary information required for the legal documentation of the vaccinations.

Of note, the same faculty members involved in the educational webinars supervised the registration stations. Community health workers and church volunteers occupied the remaining roles within the registration workflow, which aided in creating an atmosphere of familiarity and comfort for community members willing to get vaccinated in the clinics. Additionally, bilingual community health workers helped promote vaccination clinics within nearby neighborhoods in advance of clinic dates to increase attendance and to educate community members on the benefits of COVID-19 vaccination.

### 2.4. Transport of Vaccines and Patient Vaccinations

The non-Hispanic Black infectious diseases-trained pharmacist transported the vaccines and clinical supplies from the LLU Medical Center (the institution assigned the vaccines by the county) to each community vaccination clinic. The pharmacist was responsible for confirming the accuracy of each vaccine drawn by the professional student volunteers and served as the clinical preceptor for between six to eight professional student vaccinators that staffed the clinics. The pharmacist remained available for all outstanding questions from clinic volunteers and/or vaccinees.

### 2.5. First and Second Dose, and Single-Dose Vaccination Clinics

As the two-dose mRNA vaccines were the first to receive an emergency use approval (EUA) from the FDA, the Moderna^®^ vaccine was used in our initial rollout of the community vaccination clinics [11]. The first and second dose clinics were held at the same site location to reduce access barriers. After receiving their first dose of the mRNA vaccine, clinic patients were informed that the second dose mRNA clinic would take place at the same location and that they were to report back 28 days later. This date was recorded on the patient’s proof of vaccination card, as were contact telephone numbers. The patients were then contacted by the LLU ICP assigned personnel, prior to the clinic date to remind them of their upcoming second vaccination dates and the location of community clinic sites to receive the second dose. Of note, clinics using the Johnson and Johnson^®^ single-dose vaccine were initiated following its EUA approval in April 2021 [13]. A single vaccine was provided at each clinic and broadcasted to the vaccine seekers prior to the clinics. The patients were not given a preference.

## 3. Results

Descriptive data for the six community vaccination clinics, conducted between 19 February 2021, and 15 April 2021, included race/ethnicity, age, gender, and vaccination dose. All patients vaccinated were aged 18 and older. The six clinics were equally distributed across the Hispanic/Latino and non-Hispanic Black communities, (two each mRNA vaccine clinics (first and second dose) and one single-dose clinic). As shown in Table 1, a total of 1704 vaccinations were administered across six clinic sites, with 902 and 802 of those doses being delivered in the Hispanic/Latino and non-Hispanic Black communities, respectively. In both the Hispanic/Latino and non-Hispanic Black communities, individuals aged 45–64 years old were the predominant age group vaccinated, and women were more represented amongst those vaccinated. Further, the single-dose Johnson and Johnson^®^ vaccination clinic was met with a higher participation rate when compared to the two-dose mRNA vaccine clinics in both the Hispanic/Latino and the non-Hispanic Black communities.

As shown in Table 2, the mRNA (Moderna^®^) vaccine second dose return rates were 93.4% and 86.3%, respectively, in the non-Hispanic Black communities and the Hispanic/Latino communities. Of note, 41 individuals received a first dose of the mRNA vaccine during the Moderna^®^ second dose clinic held in the Hispanic/Latino community. They were not included in the return rate calculation (accommodations were made for them to receive their second vaccination). Overall, 1516 (89%) of the vaccine doses delivered in the LLU community vaccination clinics were received by non-Hispanic Black and Hispanic/Latino individuals. A total of 4% of the total vaccinees in both the LLU Mass Vaccination Clinic and the San Bernardino County Vaccination Clinics were non-Hispanic Black, while 24% and 25.6% of the clinic vaccinees identified as Hispanic/Latino for either clinic site, respectively, as shown in Table 3.

## 4. Discussion

Two years into the availability of COVID-19 vaccinations, less than 50% of eligible non-Hispanic Black and Hispanic/Latino individuals have received at least one dose of the available immunizations [14]. Consequently, individuals that are unvaccinated are 11.7 times more likely to be hospitalized with COVID-19 when compared to those that have been vaccinated; thus, amplifying the continued deleterious effects of the COVID-19 pandemic within racially and ethnically minoritized communities [14]. To this, the aim of the three-tiered approach was to collaboratively develop a community-based participatory research model and pilot an intervention that included the provision of evidence-informed vaccine education in addition to the completion of low-barrier vaccination clinics.

We effectively provided 1704 vaccine doses within non-Hispanic Black and Hispanic/Latino communities of San Bernardino County California. A higher percentage of non-Hispanic Black and Hispanic/Latino individuals were vaccinated in the community vaccination clinics when compared to the ‘traditional’ clinic model utilized by the LLU Mass Vaccination Clinic and the San Bernadino County Department of Public Health. Of note, the use of a multicomponent, community–academic approach to increase vaccination rates within Californian Hispanic/Latino communities, has been previously described by Marquez et al. [30]. The authors delivered greater than 20,000 vaccine doses in 16 weeks, mostly to Hispanic/Latino individuals, through providing education, empowering community members, and placing a low-barrier vaccine clinic in an accessible area of San Francisco [30]. Furthermore, the authors report that 90.7% of vaccinees completed both vaccine doses; therefore, emphasizing the importance in disseminating education pertaining the vaccinations and providing a consistent, unchanging location for first and second vaccine doses [30].

Additionally, our clinics showcased the importance of race concordance on communication and health outcomes [31,32]. To this, the roles in our community vaccination clinics were majority occupied by racially concordant healthcare professionals and the clinics were completed after the vaccine education webinar led by the faculty members. Thus, the increased percentage of vaccinees serviced by the community vaccination clinics is in line with previous studies that have documented the benefits of using trusted messengers to promote health initiatives [31,32]. Moreover, the role of faith-based organizations in community engagement, particularly health promotion, has been previously described [33,34]. In studies designed to address hypertension and human immunodeficiency virus (HIV) within minoritized communities, respectively, the authors report substantial benefits following the inclusion of faith leaders into the intervention [33,34]. The increases in vaccine uptake recognized in our study echo the utility of integrating faith-based organizations into community-engaged research.

Our study also highlights required differences in the tailoring of strategies to promote vaccine uptake in different racially and ethnically minoritized communities [32]. To engage the Black communities, we relied on a centralized approach, centered on our connections with the faith leaders and they primarily facilitated our entry into their communities. Nonetheless, to engage the Hispanic/Latino communities, we utilized a decentralized approach that relied heavily on community health workers to serve as the trusted entities and to facilitate our partnerships within their communities. Nevertheless, in both non-Hispanic Black and Hispanic/Latino communities, easily accessible options for vaccination are an imperative aspect for increasing uptake.

Further, the inclusion of health professions students enrolled in various LLU professional programs (pharmacy, medicine, dentistry, nursing, and respiratory therapy) as the primary vaccinators within the vaccination clinics (under the supervision of a licensed proctor) was paramount to our strategy. Although advancements in the curricula of most professional programs toward health equity content have been made, it is essential to provide students with experiential opportunities to identify their role in mitigating healthcare disparities [35,36]. To that, the vaccine clinics provide an early introduction into interprofessional collaboration, which is fundamental to creating a more equitable healthcare system. It is now a stated part of our health professional programs to facilitate and offer such experiences, providing the opportunity to expand the types of efforts described in this paper.

Nonetheless, there are several limitations to our study. First, we did not quantify the number of individuals vaccinated in the clinics that had attended the vaccine education webinars. Thus, we were unable to directly measure the influence that the vaccine education webinars had on the community vaccination clinic turnout. In the same regard, we may have underestimated the influence of our tiered, community approach as we could not quantify the number of individuals that were motivated to receive the immunization from elements of the model but were vaccinated at different sites. Further, the tiered approach was informed by identified barriers within non-Hispanic Black and Hispanic/Latino communities of San Bernardino County, CA; thus, they may not be generalizable to other races/ethnicities or other regions.

## 5. Conclusions

While COVID-19 has exposed the structural and social determinant of health inequities that burden minoritized individuals, these circumstances are not a monolith. Non-Hispanic Black and Hispanic/Latino individuals have been historically underrepresented amongst those who receive vaccinations against preventable diseases and die at higher rates [8,37,38]. Thus, identifying strategies to increase vaccine confidence and uptake is of extreme importance. To this, our described strategy of partnership between academic institutions and community/faith organizations may offer a sustainable mechanism to promote vaccine equity within non-Hispanic Black and Hispanic/Latino communities. As COVID-19 rates continue to spike, variants emerge, and vaccination protection wanes, it will be crucial to maintain these services within minoritized communities. Thus, future directions for our community–academic model will include identifying new community partners and offering live engagement opportunities. We also hope to expand the provided education to include information on other diseases that disproportionately affect minoritized groups. Moreover, as the clinics expand, we will include additional vaccinations that protect against other respiratory infectious diseases (e.g., influenza) and have declined significantly during the COVID-19 pandemic [37]. Ultimately, community-informed measures, alongside the dedication in maintaining collaborative partnerships, can have a significant impact on mitigating public health inequities observed across racially and ethnically minoritized groups.

## Figures and Tables

**Table 1:** Socio-demographic characteristics of community vaccine clinic vaccinees.

	Vaccination Clinics in the Hispanic/Latino Communities				Vaccination Clinics in the Black Communities		
		Moderna ^®^ First Dose Clinic	Moderna ^®^ Second Dose Clinic	Johnson and Johnson^®^ Single Dose Clinic	Moderna ^®^ *First Dose Clinic*	Moderna ^®^ Second Dose Clinic	Johnson and Johnson^®^ Single Dose Clinic
		*n* (%)	*n* (%)	*n* (%)	*n* (%)	*n* (%)	*n* (%)
Age	17–24	19 (7.3)	18 (6.1)	38 (10.9)	29 (11.5)	24 (10.1)	21 (6.7)
	25–44	64 (24.6)	69 (23.5)	97 (27.9)	65 (25.8)	70 (29.7)	81 (25.8)
	45–64	110 (42.3) *	133 (45.2) *	162 (46.6) *	124 (49.2) *	114 (48.3) *	156 (49.7) *
	65+	67 (25.8)	74 (25.2)	51 (14.7)	34 (13.5)	28 (11.9)	56 (17.8)
Race/Ethnicity	Black	5 (2.0)	3 (1.0)	35 (10.0)	140 (56.0) *	124 (52.5) *	144 (45.9) *
	American Indian or Alaskan Native	0 (0.0)	0 (0.0)	1 (0.3)	1 (0.4)	1 (0.4)	2 (0.6)
	Native Hawaiian	0 (0.0)	0 (0.0)	0 (0.0)	0 (0.0)	2 (0.8)	1 (0.3)
	Asian American	3 (1.2)	1 (0.3)	6 (1.7)	4 (1.6)	4 (1.7)	18 (5.7)
	Hispanic/Latino	239 (91.9) *	279 (94.9) *	268 (77.0) *	86 (34.1)	95 (40.3)	99 (31.5)
	White	12 (4.7)	10 (3.4)	33(9.5)	9 (3.6)	5 (2.1)	17 (5.4)
	Other	0 (0)	0 (0)	2 (0.6)	1 (0.4)	2 (0.8)	20 (6.4)
	Unknown	1 (0.4)	1 (0.3)	3 (0.9)	11 (4.4)	3 (1.3)	13 (4.1)
Gender	Female	157 (60.4) *	173 (58.8) *	175 (50.3) *	131 (52) *	126 (53.4) *	183 (58.3) *
	Male	103 (39.6)	121 (41.2)	173 (49.7)	121 (48)	110 (46.6)	131 (41.7)
Clinic Totals		260	294 **	348	252	236 **	314
Total of Vaccinations Provided to Each Community	902				802		
Grand Total	1704						

* Indicates the majority % of vaccinees; ** Totals include individuals that received first dose at second dose clinic.

**Table 2:** Community vaccination clinic second dose return rate (mRNA vaccine).

mRNA Vaccine Dose (Moderna^®^)	Vaccination Clinic Targeted Minoritized Group	
	Hispanic/Latino Community Clinic	Non-Hispanic Black Community Clinic
First Dose Clinic	260	252
Second Dose Clinic	243	219
Return Rate Total Percentage	93.4%	86.9%

**Table 3:** Race/ethnicity demographics of LLU Community Vaccination Clinics compared to the LLU Mass Vaccination Clinic and the San Bernardino Countywide vaccination efforts [26].

Race/Ethnicity	LLU Community Vaccination Clinic	* LLU Mass Vaccination Clinic	* San Bernadino Department of Public Health Countywide Vaccinations
	*n* (%)	*n* (%)	*n* (%)
Black American	451 (26.5)	2579 (4)	50,578 (4)
American Indian or Alaskan Native	5 (0.03)	353 (0.5)	0 (0)
Native Hawaiian	3 (0.02)	203 (0.3)	0 (0)
Asian American	36 (2.1)	13,527 (21)	74,544 (5.9)
Hispanic/Latino	1066 (62.6)	15,494 (24)	561,170 (44.7)
White	85 (5)	27,680 (43)	210,949 (16.8)
Other	26 (1.5)	2251 (3)	320,852 (25.6)
Unknown	32 (1.9)	2719 (4)	36,877 (2.9)
Grand Total	1704	64,806	1,254,970

* The LLU Mass Vaccination Clinic and San Bernardino County Vaccination include all vaccination doses provided up until 31 March 2022.

## Data Availability

The data that support the findings of this study are available from the corresponding author, J.C.A.-M. upon reasonable request.
